# NICU discharge preparation and transition planning: guidelines and recommendations

**DOI:** 10.1038/s41372-022-01313-9

**Published:** 2022-02-14

**Authors:** Vincent C. Smith, Kristin Love, Erika Goyer

**Affiliations:** 1 Boston Medical Center, Boston, MA USA; 2 National Perinatal Association, Lonedell, MO USA; 3 National Perinatal Association, Austin, TX USA

## Abstract

In this section, we present Interdisciplinary Guidelines and Recommendations for Neonatal Intensive Care Unit (NICU) Discharge Preparation and Transition Planning. The foundation for these guidelines and recommendations is based on existing literature, practice, available policy statements, and expert opinions. These guidelines and recommendations are divided into the following sections: Basic Information, Anticipatory Guidance, Family and Home Needs Assessment, Transfer and Coordination of Care, and Other Important Considerations. Each section includes brief introductory comments, followed by the text of the guidelines and recommendations in table format. After each table, there may be further details or descriptions that support a guideline or recommendation. Our goal was to create recommendations that are both general and adaptable while also being specific and actionable. Each NICU’s implementation of this guidance will be dependent on the unique makeup and skills of their team, as well as the availability of local programs and resources. The recommendations based only on expert opinion could be topics for future research.

## About the guidelines

The foundation for these recommendations is based on existing literature, practice, and available policy statements. Given the range of topics we cover, there are some situations where there is no published literature specific to a recommendation. In some situations, we relied on the lived experiences of families and providers to inform our recommendations. While there may not be supporting references for some of these recommendations, all of the recommendations are based on expert opinion and consensus and the readers are requested to note this issue while adapting them into their practices, if they choose to. The recommendations based only on expert opinion could be topics for future research.

Our guidelines are divided into the following sections:

- Basic Information
- Anticipatory Guidance
- Family and Home Needs Assessment
- Transfer and Coordination of Care
- Other Important Considerations

Each section includes brief introductory comments, followed by the text of the guidelines and recommendations in table format. After each table, there may be further details or descriptions that support a guideline or recommendation.

## Using the guidelines

It is impossible to create a comprehensive discharge preparation and transition planning program that will work for every family in every NICU setting. Rather, what we propose are guidelines and recommendations that focus on content and process. We strived to create recommendations that are both general and adaptable while also being specific and actionable. Each NICU’s implementation of this guidance will be dependent on the unique makeup and skills of their team, as well as the availability of local programs and resources.

## Basic information

Discharge planning is the process of working with a family to help them successfully transition from the NICU to home. To this end, each family will need to participate in a comprehensive discharge planning program that has been tailored to their and their infant’s specific needs. The first section is basic information and is meant to emphasize content that every family will need, without taking into account each family/infant’s specific needs.

In preparing for discharge, your team will have to set clear criteria for what each family and infant need to accomplish to be ready to transition from the NICU to home. The NICU team should work with the family and confirm that the family understands the NICU discharge planning process. It is important that families understand that it is difficult to plan for a specific discharge date because discharge readiness is often conditional (e.g., the infants has no further spells, is able to gain weight, pass a car seat test, etc.) The fluid and uncertain nature of discharge readiness can be a source of frustration for families. To help minimize frustration and avoid misunderstandings, it is important to have consistent messaging, emphasizing that there can be wide variations in when an infant is discharged based on clinical indications and medical opinions.

### Discharge educational content

The discharge educational content is the foundational curriculum for each family in the NICU. Each family will need to have infant care knowledge and skills to be able to provide care at home for their infant. The topics covered in this section include infant care skills demonstration, family comprehension assessment, timing of discharge education, and some elements to support family education (see Table 1).

**Table 1:** Discharge education.

DISCHARGE EDUCATION	
RECOMMENDATION	SUPPORTING REFERENCES
DISCHARGE EDUCATION CONTENT	
Communicate to the family the skills that need to be mastered prior to discharge and the expected timing of discharge.	[1, 2, 7, 14, 16–21]
Families need to have demonstrated appropriate technical infant care skills and knowledge prior to discharge. Common infant care topics that families need to understand prior to discharge include the following:	
• How they will safely feed their baby. ◦ How to support feeding at the breast and using a bottle. ◦ How to mix formula or increase calories in breast milk as indicated. ◦ How to pump and store breast milk. • How to bathe their baby. • How to dress their baby for the weather and for sleep. • How to diaper their baby and what is a typical number of bowel movements or wet diapers for their baby to have each day. • Preparing a crib, bassinette, or bed at home for the infant and creating a safe sleep environment. • Circumcision and umbilical cord care as indicated. • Infant home medication information: ◦ Medicines (including vitamins and other supplements) the infant will take when they are at home. ◦ Medical indication for each medication. ◦ Administration instructions for each medication. ◦ What to do if the infant misses a dose of the medication. • Recognizing fever and other potential signs of illness and who to call with medical questions/concerns. ◦ When to call the pediatrician. ◦ When to call 911 or emergency services. • How to protect the infant from infections. ◦ Handwashing and hygiene instructions. • Safe use of infant-related technology. • Importance of having a medical home for the infant with primary care providers who are familiar with the needs of infants who have been in a NICU. • When and how to use a bulb syringe. • Infant-specific cardiopulmonary resuscitation (CPR). • When and how to do tummy time. • Importance of vaccinations for infants and their caregivers. • Understanding infant enrollment in special programs for preterm infants and infants with special medical or developmental needs, as appropriate. • Preparing the home environment. • Arranging for help that the family may need at home.	[1–4, 6, 8, 14, 16, 22–24]
Discharge instructions should facilitate family’s understanding of major and/or significant diagnoses.	[1, 5, 22]
Discharge instructions should include the following information: • Primary care follow-up location, date, and time. • Follow-up medical specialist appointments that have been scheduled, as well as appointments that are needed but have not yet scheduled. ◦Medical indication and/or rationale for each medial specialist service follow up. • Advice about who to contact in case of an unanticipated occurrence.	[1–4, 6, 14, 16, 18, 20, 22, 25]
FAMILY PREFERRED EDUCATIONAL MODALITY	
Identify how the family prefers to receive and review information. Their preferred method should be used for the discharge planning process.	[9]
Offer remote discharge teaching if a caregiver is located in a different physical location, making use of technology with a preference for video instead of just audio (e.g. FaceTime, Zoom, Skype, etc.).	[18]

FAMILY COMPREHENSION	
Invite family members’ questions. But instead of asking, “Do you have any questions?” say, “Did I leave something out?” or “Does that make sense?” Communicate clearly that you expect they will have questions, both now and in the future. Let them know who can help answer those questions.	[5, 14]
Confirm the family’s comprehension of the infant’s diagnoses and conditions and their knowledge of medical follow-up appointments by inviting the family to repeat or “teach back” their understanding.	[1, 3, 5, 14, 26]
Ensure the family’s comprehension of infant care skills, confirmed by return demonstrations of their knowledge. Encourage families to demonstrate by suggesting, “Now you try it”.	[1, 5, 8, 14, 19–21]
TIMING OF DISCHARGE EDUCATION	
Families’ technical infant care skills and knowledge discharge preparation should occur based on the family’s availability, regardless of time of day. When possible, have at least two caregivers that are familiar with the infant care skills and knowledge.	[4, 14, 18]
Establish that the family feels comfortable with their ability to provide care at discharge. This may require changing the baby’s existing care schedule to accommodate parent availability and/or an overnight stay.	[6, 14, 17]
FAMILY EDUCATION SUPPORT	
Some families may want to record infant care skill demonstrations for later reference or to show a family member who was not present during the demonstration. Follow hospital protocol to allow video recording as appropriate. Note: if there is no policy, it may be necessary to create one to support a family’s use of recording as part of the education process.	[5]
Supplement discharge skill demonstrations and discharge education in the family’s preferred learning style and language (i.e. written, visual, live demonstration, or recorded) to reinforce instruction and increase knowledge retention. Allow multiple educational opportunities and skill demonstrations. Give families adequate time to process information and ask questions. Accommodate the family when additional consultation or a review is requested.	[1, 4, 5, 18]

### Discharge planning tools

This section discusses resources that could be helpful in supporting families’ discharge preparation. Some elements discussed include the discharge summary, NICU hospital course roadmap, and discharge planning folder. In this context, the NICU hospital course roadmap is intended to be a strategic plan that defines a goal or desired outcome and includes the major steps or milestones needed to reach it (see Table 2).

**Table 2:** Discharge planning tools.

DISCHARGE PLANNING TOOLS	
RECOMMENDATION	SUPPORTING REFERENCES
DISCHARGE SUMMARY	
Discharge summaries should at minimum include all the following items: • Infant’s name in the hospital (and after discharge, if they are different). • Discharge diagnoses. • Condition at discharge. • Discharge physical exam findings (highlighting any abnormal physical findings). • Discharge medications and administration instructions. • Home feeding plan. • Newborn hearing screen results, any follow-up screening needed. • Newborn screening dates sent and (if known) any abnormal results. • Car seat screening results. • Immunizations administered, any immunizations recommended but not yet administered. • Pending test or lab results that need to be followed up on. • Prognosis if guarded. • If indicated, include the infant’s medical equipment needs (e.g., oxygen, gastrostomy tube, etc.). • Any known pertinent social, family, or medical history. • Community service program referrals made or recommended (e.g., community health nursing agencies, early intervention services) and any counseling opportunities available to the family. • Any tasks to be completed (e.g., follow-up appointments or test that were recommended but not yet scheduled). • Interpreter and/or communication needs. • Any referrals to resources for specific diagnoses. • Community resources (e.g., counseling services, mental health support, substance dependency treatment, visiting nurses, financial resources, etc.).	[1, 4–6, 16, 26]
Discharge summary should be formatted from a structured template with section headings.	[1, 5, 25]
Discharge summary would, ideally, be translated into the family’s preferred language.	[1, 27]
As part of the discharge process, provide families with copies of the final discharge summary and directions on how the family can get an official copy of the medical record, if they would like.	[1, 5]
Provide at least two copies of the discharge summary (one for the medical home and one for the child’s family that they can share with home visiting or emergency department services as needed). If the infant is seen by medical specialists, either provide the specialists with copies of the discharge summary directly or provide the family with copies of the discharge summary to give to the specialists.	
NICU ROADMAP	
Have a roadmap or equivalent visual schematic that outlines the time span from the birth and NICU admission to NICU discharge. On the roadmap, include following: • For the family, individualization of discharge educational goals/task and a suggested timeline to complete the items. • For the infant, medical milestones for the infant to achieve, relevant to the infant’s gestational age and unique developmental progression. • Identification of short- and long-term follow-up needs of the infant and plans for the transition to pediatric care.	[1, 4]
DISCHARGE PLANNING FOLDER	
Provide a discharge planning and education folder, to be used during the discharge planning process to guide the family through the topic areas and tasks they and their baby must achieve for discharge. It may contain a checklist and other educational materials that allow the family and staff to track the family’s progress with discharge preparation.	[1, 17]

WRITTEN DISCHARGE INFORMATION	
All printed materials given to families should be written in a manner that is simple, clear, concise, and devoid of medical jargon. This aids understanding and decreases confusion. Written materials should be reviewed to ensure that they meet health education standards. When possible, include written and illustrated materials in the family’s preferred language to support and reinforce verbal instruction.	[1, 5]
SUPPLEMENTAL DISCHARGE EDUCATIONAL MATERIALS	
Parents should be provided with materials (e.g., binders, folders, videos, websites) to supplement bedside infant care skill knowledge and education.	[1, 4, 5, 22]
JOURNAL	
Some families benefit from having a journal to document their discharge education, describe experiences, and serve as a place to keep track of their observations, questions, and concerns.	

### Discharge planning team

In addition to the family, the discharge planning team may include clinical nurses, physicians, mid-level providers (e.g., neonatal advance practice nurses and physician assistants), discharge coordinators, discharge planners, case managers, psychologists, social workers, and other providers as needed. Because the personnel available in each NICU vary, it is important to focus on the function and not the title (See Table 3).

**Table 3:** Discharge planning team.

DISCHARGE PLANNING TEAM	
RECOMMENDATIONS	SUPPORTING REFERENCES
INFANT CARE GIVERS	
At the beginning of the discharge planning process, identify the people who will be primary caregivers for the baby and ask how willingly that responsibility is assumed. Those individuals, and any others that the family prefers, will be the primary recipients of the NICU discharge preparation program.	[3]
CONSISTENT NURSING PROVIDER	
Families benefit from having consistent bedside nursing that allows the nurse and family to become familiar with each other. Some units are able to achieve more consistency by having primary nursing and/or nursing teams for each infant.	[1, 14, 23, 28]
FAMILY SUPPORT PEOPLE	
Some families may benefit from having more support people than just the parents participate in the discharge planning process. During the assessment process, allow families to invite their preferred support people to participate using their method of choice (i.e., remote, in person, etc.). Some examples of support people include, but are not limited to, extended family; partners; close friends; church, temple, or spiritual leaders; doulas, midwives; home health aides; community leader, etc.	[6, 14, 15, 24, 29]

DISCHARGE COORDINATOR/DISCHARGE PLANNER/CASE MANAGER	
This individual(s) is responsible for ordering durable medical equipment, helping arrange follow-up appointments, and coordinating services for after discharge (e.g., physical or occupational therapy, home health care, private duty nursing, etc.). Also, this individual is responsible for assisting the family with contacting the fire department and/or electric company and creating a plan if they lose electricity at home.	
SIBLING RESOURCES	
Siblings may need age-appropriate support to understand the NICU experience, the babies’ medical and developmental needs, and the resulting changes in their home life. Positive interventions could include sibling support programs, activities, classes, and/or resources. When able, use Child Life specialists to help with sibling support programming.	

Often the physician’s role in discharge planning is to ensure the baby has met the physiological discharge criteria and that the family has completed the comprehensive discharge planning program. The physician or designee is generally responsible for providing a warm handoff to the medical home that will assume the care of the infant and family after they leave the NICU.

Primary care providers and discharge coordinators provide ongoing technical and emotional support for families. NICU staff who have established rapport with the family play an important role as the family transitions from NICU to home [1].

Members of the nursing team who have worked most closely with the family during their baby’s hospitalization will be the best prepared to help the family as they plan for discharge. They not only know the infants’ health history, they also have experience working with the family and may have insights about parents’ perceived strengths and unique vulnerabilities [1].

NICU social workers have a wide range of responsibilities. Their role is dependent on what other resources are available in each NICU. Some families will not be clear about the role of the NICU social worker and may have misgivings about their involvement. This is especially true for families who have experienced the negative effects involvement with the child welfare system. It is helpful to clearly establish the role of social workers in the NICU setting. Providing all families with written information about available social services and supports and the role of the NICU social worker is advisable early in a family’s NICU stay.

NICU psychologists support families by providing positive therapeutic interventions, emphasizing techniques that support and facilitate parent-infant bonding. They can administer appropriate mental health screenings and provide timely referrals, in support of the families’ health and wellbeing. If your NICU does not have a psychologist, others may assume these roles if they have proper training and expertise.

### Discharge planning process

For these guidelines, discharge planning process tries to address some who, when, and where questions. This section provides some guidance on discharge planning timing, discharge planning meetings, discharge planning goals, and educational philosophy (see Table 4).

**Table 4:** Discharge planning process.

DISCHARGE PLANNING PROCESS	
RECOMMENDATIONS	SUPPORTING REFERENCES
DISCHARGE PLANNING TIMING	
Discharge planning should begin at admission and continue throughout the infant’s hospitalization.	[2, 3, 8, 14, 17, 18, 27]
DISCHARGE PLANNING MEETING	
The initial discharge planning meetings should integrate the family in discharge planning process and continue with step-by-step planning as discharge approaches. • Routinely include the family in discharge meetings, normalizing the process and helping to ensure consistent messaging to the family. This creates a shared vision for discharge planning between the family and staff. • Allow space for the family to voice their comments/concerns about the discharge plan, then respond to their needs. • Give the family enough advanced notice to plan to attend the meetings. Be flexible/family-friendly with the scheduling of meetings (e.g., day or night) to maximize family participation. • When possible, face-to-face discharge planning meetings are preferred. But allow the family the option of remote participation. • Part of the discharge planning meeting is to ensure that the family has a good understanding of where they are in the discharge process, confirm the educational goals, and verify the discharge criteria to allow the family time to do home environment preparation, obtain home supplies, arrange help at home, and schedule follow up appointment arrangements. • Some families will want to include their preferred support network in discharge planning. Staff should accommodate the needs of the family and not limit the meetings to only the parents, unless that is the family’s preference.	[1, 2, 14, 15, 18]
DISCHARGE PLANNING GOALS	
Parents should be an integral part of the multidisciplinary team. This team collaborates to create a timeline of discharge goals, educational objectives, and specific technical skills that must be attained.	[1, 4, 14, 19, 20]

EDUCATIONAL PHILOSOPHY	
Discharge education strategies should be culturally-responsive and tailored to meet the specific needs of the family.	[1, 2, 4, 8]
Medical teams should utilize the tenets of Family-Centered Care: • Dignity and Respect. • Information Sharing. • Family Participation in Care. • Family Collaboration.	[1, 2, 21, 23]
Ensure that staff members provide a consistent, uniform message when communicating the discharge instructions.	[5]
Education modalities should be flexible to meet the needs of the family. • Plan ahead (more lead time may be needed because of the potentially more complex logistics). • Be flexible with timing to allow for scheduling with both parents if they would both like to participate. • If one of the caregivers is not able to be present, offer remote discharge education, making use of video technology (e.g., FaceTime, Skype, Zoom, etc.) when possible and audio when necessary.	

## Anticipatory guidance

Anticipatory guidance—in the context of NICU discharge preparation and transition planning—refers to helping the family develop a realistic idea of what their life will be like with their infant. This means in the immediate future following discharge as well as over their life course.

Work with the family to develop a realistic understanding of how to care for their child at home (see Table 5). Some ideas to reach this goal include the following:

- Create a first month at-a-glance calendar that contains important daily life events (e.g., feeding times, medication administration times, appointments, etc.).
- Discuss the number of provider appointments and indicate which will be appointments in the home versus which will be an office/hospital visit.
- Explain that the baby and family will need time to adapt to their new environment and there may initially be some changes in the child’s behaviors, including feeding and sleeping behavior.

**Table 5:** Home and family life.

ANTICIPATORY GUIDANCE	
RECOMMENDATION	SUPPORTING REFERENCES
HOME AND FAMILY LIFE	
Provide a realistic idea of what life will be like during the immediate and more longer-term period following discharge including: • Expected number and types of physician visits for routine infant health care and illness or specialized care. • Anticipated infant developmental milestones and the range of age when they may achieve these milestones. • Anticipated and potential infant developmental and/or growth-related challenges. • Activities expected during the first year of the infant’s life to help their baby grow and develop both physically and socially.	[14, 18, 22, 24, 25, 27, 30]

Prior to discharge, families should understand typical, expected infant behaviors including feeding, voiding and stooling, and sleeping patterns. Families also need to understand atypical infant behaviors, as well as signs and symptoms of potential illness (see Table 6).

**Table 6:** Infant behavior.

ANTICIPATORY GUIDANCE	
RECOMMENDATION	SUPPORTING REFERENCES
INFANT BEHAVIOR	
Explain typical versus atypical and/or concerning infant behaviors and help parents understand their baby’s cues.	[1, 2, 4, 17, 18, 22–24]

It can be very stressful coping with a crying baby, especially when the caregiver is sleep deprived. Work with the family to manage their anticipated exhaustion and focus on strategies that reduce the risks of harm to the infant, including having a rescue/respite plan when the caregiver has reached their limit (see Table 7).

**Table 7:** Coping with a crying infant.

ANTICIPATORY GUIDANCE	
RECOMMENDATION	SUPPORTING REFERENCES
COPING WITH A CRYING INFANT	
Families must be taught how to soothe their crying infant and strategies for coping when a baby is difficult to soothe. Families should understand the risks for Shaken Baby Syndrome and the harm caused by shaking, slamming, hitting or throwing an infant.	[1, 2, 6]

Despite the best of circumstances and planning, sometimes emergencies or unanticipated problems will arise. Prepare families to respond in an emergency (Who, What, Where, When, How, and Why). Emergency planning can include an assessment of perceived acuity and an appropriate response (e.g., calling emergency services or 911 versus the contacting the pediatrician versus waiting until the next scheduled pediatric visit). To assess perceived acuity, some families may benefit from using a visual graph of green (wait for the next scheduled pediatric visit), yellow (call the pediatrician right away), or red (call 911 or emergency services). Encourage the family to learn how to use the pediatric patient portal if you have one and if they have access to a computer and/or smartphone (Table 8).

**Table 8:** Emergency planning.

ANTICIPATORY GUIDANCE	
RECOMMENDATION	SUPPORTING REFERENCES
EMERGENCY PLANNING	
Ensure the family knows where to go or who to call if there is an urgent care need or medical emergency.	[1, 3, 4, 22]

Many families may not be aware of mental health issues that can typically follow a NICU experience or traumatic birth. It is valuable to assist families understand expectations for caregiver emotional responses (see Table 9). Staff and families both need to understand post-traumatic stress disorder is not solely a military combat disorder, rather it is a normative reaction to extraordinary circumstances. To address mental health issues, provide families with resources that include interventions and strategies they can use in the moment and locally available support resources (i.e., national hotlines, neighborhood support groups).

**Table 9:** Parental mental health.

ANTICIPATORY GUIDANCE	
RECOMMENDATIONS	SUPPORTING REFERENCES
PARENTAL MENTAL HEALTH	
Parents should be advised about typical, anticipated mental health issues. Pediatricians play an important role and need to be aware of the unique mental health needs of families.	[1, 2, 4, 10, 24, 31–33]
All families need to be aware of the effects of trauma and the potential for post-traumatic stress. Facilitate family’s understanding of possible post-discharge emotional reactions to their infant’s birth and NICU stay.	[9, 10, 24, 31–33]

The costs associated with a NICU stay continue long past a family’s discharge. Families will not receive the final bills until the baby has been home for some time. Many families will be unprepared to navigate all the financial burdens associated with NICU hospitalization and follow-up care on their own. Offering families clear anticipatory guidance to help families understand how to manage the costs. Offer every family financial counseling and resources. Help them apply for assistance. Provide them the referrals and letters of medical necessity that can help remove barriers to the care their infants need. Each family should be offered this service regardless of perceived means. Social work or case management are often the best prepared to engage families in discussions about finances (see Table 10).

**Table 10:** Paying for a NICU stay.

ANTICIPATORY GUIDANCE	
RECOMMENDATIONS	SUPPORTING REFERENCES
PAYING FOR A NICU STAY	
Arrange for the family to meet with social worker or case manager about potential financial burden associated with NICU and follow up care.	[2, 6]

## Family and home needs assessment

This section discusses family and home needs assessment to inform discharge planning.

Develop a process for assessing families’ needs and connecting them with appropriate community resources. A transition plan must include the medical, physical, psychosocial, and mental health needs of both the family and infant. All families should be screened upon admission to NICU for social determinates of health and risk factors (see Table 11). Address risk factors early through referral to community partnerships and social work services for needed services (i.e., housing, finance, etc.). Each NICU should have a readily available, easily accessible community referral guide with information about programs and agencies supporting families. This should be a living document; new resources should be added as they are made known (Table 12).

**Table 11:** Family and home needs assessment process.

FAMILY AND HOME NEEDS ASSESSMENT PROCESS	
RECOMMENDATIONS	SUPPORTING REFERENCES
TIMING OF ASSESSMENT	
Help families understand and address social determinates of health. All families should be assessed for risk factors upon admission to NICU and again as part of the discharge planning process.	[2–4, 34]

**Table 12:** Family and home needs assessment content.

FAMILY AND HOME NEEDS ASSESSMENT CONTENT	
RECOMMENDATIONS	SUPPORTING REFERENCES
FAMILY LIVING ARRANGEMENT ASSESSMENT	
Assess where the family is currently living and where they will be living after discharge. Ascertain if there are special considerations related to location that could affect discharge planning (e.g., rural setting or limited local resources).	[3, 6, 16, 27]
HOME SUPPLIES ASSESSMENT	
Prior to discharge, confirm the family has the supplies and equipment they will need to provide care for their infant at home. This includes, at minimum, confirming they have a food, diapers, crib/bassinette, safe sleep environment, and a car seat for the infant.	[3, 4]
HOME ASSESSMENT	
The home assessment should confirm secure housing for the family and gauge basic essentials such as safe/adequate water, electricity, heat, cooling, smoke/carbon monoxide detectors, and if needed space for medical equipment. When appropriate, ask about the physical space in which the family will be living to make sure it can accommodate appropriate home medical equipment.	[3, 16, 27]
TRANSPORTATION ASSESSMENT	
Determine if the family has any problems with transportation that would adversely affect their ability to attend medical follow-up appointments. With the family’s permission, communicate this with community providers. Offer information on medical transportation.	[2, 3, 9, 16, 27]
CHILD CARE NEEDS	
Explore with the family their plan for child care after discharge from the NICU. Help them communicate their babies’ needs with caregivers.	[2, 3, 9, 16, 27]
NUTRITION ASSISTANCE	
Determine if families meet criteria for social programs including Special Supplemental Nutrition Program for Women, Infants, and Children (WIC), Supplemental Nutrition Assistance Program (SNAP) (formerly referred to as food stamps), Supplemental Social Security Income (SSI), SSI Disability, etc.	[2]
SOCIAL SUPPORT NEEDS	
Evaluate what social supports the family has in place (or anticipates will be in place) at discharge as well as what supports they may still need. Also, ask how the family feels about the receiving social supports.	[2, 3, 9, 16, 27]
FAMILY COPING STYLE	
Learn about the family’s coping habits and styles. Offer supportive resources.	
PARENTAL MENTAL HEALTH	
Assess parents for mental health complications in the NICU and incorporate the results into the discharge planning, This is especially important for those with a known history of mental health issues, including postpartum depression (typical and atypical), anxiety, and post-traumatic stress.	[2, 3, 6, 9, 14, 15]
Request a mental health assessment if there is concern about the parents’ bonding or attachment with the infant. This should be informed by parent report and based on the observed behavior. Provide parent-infant mental health support.	[9]
SOCIAL OR SAFETY CONCERNS	
Develop safety plans in collaboration with the family when there are social and/or safety concerns.	[2]
Assessment of the family should include screening for interpersonal violence and parental substance misuse.	[2]

The NICU staff who conducts the family needs assessment may vary due to NICU personnel composition. In general, social workers or other appropriate staff (e.g., psychologist, child life specialist, case manager, discharge coordinator, etc.) may conduct the family needs assessment during the first few days of admission and prior to discharge, with a potential reassessment as needed [2–5].

It is preferred to use a multidisciplinary team (that includes nursing, social work, case management) to assess the family’s needs [2, 5, 6]. This must be a collaborative process with the family and one where the family is the driver and owner of the assessment process. Prior to the assessment, discuss with the family what they are being assessed for, what the process will entail, and how the information will be used [3]. The person doing the assessment needs to be aware of their own explicit and implicit biases and respect the family’s right to disclose or not disclose information during the assessment process.

Some families will be considered high-risk and may need extra attention as part of the discharge planning process. Some examples of high-risk families include but are not limited to the following: families where there is active substance use or history of substance misuse, those who received inadequate prenatal care, teenage parents, those experiencing interpersonal violence, families experiencing housing or food insecurity, families where there is significant relationship instability, those with diagnosed or undiagnosed mental health conditions (especially anxiety or depression), families with limited socioeconomic resources, caregivers with low functional health literacy, those where a family member is incarcerated, and transient or migratory families [1–3, 7–13]. The reason a family is high-risk and how that information was discovered should be treated as private and sensitive information. This means that access to the information should be limited to those who need to know. To the extent possible, there may need to be an assurance of professional confidentially so that families feel comfortable disclosing information. If information can be used to potentially cause harm, such as a disclosure of substance use, families should be advised of the risks of disclosure and every effort should be made to limit the harm that the sharing of this information may lead to. If they are going to provide ethical care, staff should be aware of their own explicit and implicit biases and try to minimize their influence on the family and home assessment process.

A documented system of early intervention referral and communication with community resources is needed for babies going into foster or custodial care.

Always start by asking permission and explaining the purpose of doing an assessment or screening. A sample introduction could be, “I‘d like to ask you more about your family and who’s helping you already so that we can plan next steps together.” Some other open-end questions include:

- Where are you currently living? Is this where you will be living after discharge or will you be moving?
- Who lives in the home with you? Who stays with you regularly? Who visits?
- Are there any family members (e.g., extended family) or other support people who live near you?
- Who are the support people that your family relies on?
- Is there anything that the NICU staff should understand about your family to better serve you?
- Are other children in the family receiving any services or supports? Do they have any unmet needs?
- Who contributes to your family financially and economically? Who helps meet your needs?
- How is your family’s health care covered and paid for?

Parental mental health conditions are common and treatable, but may be missed if not specifically assessed. Mental health issues may look different in postpartum parents than in their partners/spouses. Mental health manifestations may also vary based on cultural or social context. When screening a family, be sure to use evidenced based cut off scores for the individuals being screened (e.g., partners, those impacted by racism, etc, [9, 14, 15]. Families with limited English proficiency may require a more specific mental health assessment and/or support [9, 14, 15].

Every family who requests mental health support should receive mental health support, regardless of the scores on their assessments.

## Transfer and coordination of care

This section discusses transfer and coordination of care from NICU providers to community providers and the medical home (see Table 13).

**Table 13:** Transfer and coordination of care.

TRANSFER AND COORDINATION OF CARE	
RECOMMENDATIONS	SUPPORTING REFERENCES
PRIMARY CARE INVOLVEMENT	
When discharge planning begins, the family should identify the primary care provider/practice that will be providing follow-up care for the infant. A primary care provider needs to be chosen prior to the infant being discharged. If the family has not chosen a primary care provider, the NICU team can help them make this selection.	[1, 3, 6, 12]
If not already involved, the NICU team should provide sign out to the infant’s pediatrician at the time of discharge.	[4, 19]
PRIMARY CARE CONTACT	
Within 48 hours of discharge, contact the medical home and make them aware of the history of the infant’s hospitalization and the plan for their discharge. This can be done by a telephone call, text message, fax, or email. When possible, provide important contact information to the medical home. Invite and encourage ongoing communication about the infant’s health history and hospitalization. At minimum, include the following information in this communication: • Infant’s name in the hospital and after discharge (if they are different). • Medical diagnoses. • Discharge medications and administration instructions. • Results of major procedures (e.g., sleep studies, modified barium swallows, bronchoscopy, etc.). • Test results and pending tests. • Follow-up appointments arranged and those that need to be scheduled. • Interpreter and/or communication needs. • Connections to resources for specific diagnoses or special needs (e.g., Trisomy 21, Multiples of America, etc.). • Family needed resources (e.g., counseling services, mental health, financial resources, etc.).	[5, 6, 8, 22, 26]
With complex medical or social situations prior to the infant being discharged, it is helpful to get the primary care provider involved/updated as well as collaborating on the development of a new (or commenting on an existing) plan for follow-up care. A warm handoff is preferred for complex medical and/or social situations. If social worker (or equivalent) is involved, it is preferred for them to give a warm handoff to the social worker from the primary care facility.	[3–6, 8, 19]
The discharge summary should be provided to the medical home, preferably on the day of discharge but as close to discharge as possible. This summary should be delivered no more than one week after NICU discharge. Confirm that the summary has reached the intended providers.	[5]
NICU CONTACT WITH THE FAMILY AFTER DISCHARGE	
A NICU representative (preferably someone with medical expertise such as a nurse, patient navigator, discharge coordinator, mid-level provider, social worker, etc.) will call the family within a few days after discharge to assess their understanding of the following: • discharge instructions. • feedings and how to mix the feeding. • medications and medication administration instructions. • follow up appointment dates/times and reason for appointment. During the call, the NICU representative may also inquire about the following; • general well-being of infant and family. • any anticipated or unanticipated issues/challenges that have arisen. • referrals/appointments needed that have not been made.	[5, 8, 22, 26, 29]
PARENTAL MENTAL HEALTH	
Assess the family for mental health concerns in the NICU and as the family transitions to resources in the community. Because there is variability as to when mental health issues manifest, the assessment may need to occur more than once.	[2, 3, 9]

COMMUNITY RESOURCES	
As part of the family assessment, determine which community resources, family supports, and services are needed, what the family’s preferences are for community supports, and what is available in their community.	[3, 5, 8, 29]
Maintain a robust, updated, comprehensive list of available community resources (e.g., Healthy Start, Early Intervention, Nurse Family Partnerships, etc.). When making a referral to a program, include the appropriate cultural information in the referral.	
In states where applicable, request an initial early intervention assessment and an Individualized Family Service Plan (IFSP) be completed while infant is still in NICU. This promotes a relationship of trust, potentially decreasing barriers to follow-up care.	[3, 4]
Neonatal Therapists (Occupational, Physical, Feeding, and/or Speech and Language Therapists) should be part of the referral process for early intervention services.	[3]
Assess every family for Part C* of Individuals with Disabilities Education Act (IDEA) eligibility and make a referral to qualifying families prior to discharge. See Eligibility. Section 612 of the IDEA; 20 U.S.C. 1434. *Individuals with Disabilities Education Act (IDEA) is a law that makes free and appropriate education available to eligible children with disabilities. Part B of the law covers preschool age children, and Part C covers infants and toddlers. Part C of *IDEA is a federal grant program that assists states in operating a comprehensive statewide program of early intervention services for infants and toddlers with disabilities (from birth through age 2 years) and their families*.	
Upon admission, begin to identify the needs of infants with complex medical issues and start creating a care plan for the family. Do the same for babies with special developmental needs and families with special social needs. Begin coordination with relevant community partners well ahead of the infant’s discharge.	[3, 4, 8, 28]
COMMUNITY NOTIFICATION	
When medically indicated, confirm that the family understands the need and has a plan to notify local authorities (e.g., local emergency, fire, and police departments) about the presence oxygen in the home and the potential need for assistance during power outages.	[3]

Peer-to-peer support programs have been shown to be very effective and could be assets to every NICU. After discharge from the NICU, many families would benefit from being connected to a community-based, peer-to-peer support program.

## Other important considerations

This section discusses some important topics to consider when doing discharge planning. We are mindful of families who are: limited English proficient, active military, lesbian, gay, bisexual, transgender, queer, intersex, and asexual (LGBTQIA+) headed, disabled, and/or culturally and/or philosophically distinct in ways that need to be considered in NICU discharge transition planning.

### Families with limited English proficiency

This section focuses on discharge planning with families who have limited English proficiency (see Table 14).

**Table 14:** Families with limited English proficiency.

FAMILIES WITH LIMITED ENGLISH PROFICIENCY	
RECOMMENDATIONS	SUPPORTING REFERENCES
INTEPRETER USE	
Certified medical interpreters should be used for all discharge education and the discharge planning meeting, with the order of preference being in-person, video, or phone. Discharge materials should be delivered in the family’s preferred language and communication mode.	[12, 13, 35]
Provide a medical interpreter when any primary caregiver has limited English proficiency. Family members may not be able to accurately interpret for each other, especially about medically complex concepts.	[5, 12, 35]
Plan ahead when using an interpreter. Additional time is needed to coordinate interpreter availability, family needs, and time needed for the team to process information, reflect, and consider additional questions.	[5]
Family comprehension of the discharge education and awareness of scheduled medical follow-up appointments should be confirmed by return demonstrations of their knowledge with interpreters.	[5, 12, 35]
FAMILY MEMBER USED AS AN INTERPRETER	
Family members should only be used to help interpret if a certified medical interpreter is not available or in an emergent situation. A minor should not be used as a family interpreter. After a family member has been used as an emergency interpreter, a certified medical interpreter should be brought in as soon as feasible to verify the family’s understanding of the information.	[12, 13, 35]
PATIENT-RELATED INFORMATION	
Provide families with discharge materials in their preferred language. These materials should be written in a manner that is simple, clear, concise, and devoid of medical jargon to aid understanding and decrease confusion.	[4, 5, 12, 13]
Translate patient-related information and medical records into the family’s preferred language or mode of communication by medically certified translators.	[12, 13]
COMPUTER TRANSLATION SERVICES	
Items that are translated via a computer translation service should be verified by a certified medical interpreter for clarity and accuracy.	
HOSPITAL NAVIGATION	
Develop a plan, note, card for families to use to identify themselves as having a child currently in the NICU. Some families may have difficulty accessing and navigating the hospital because they are unable to communicate with security/front desk staff or read signs.	[12]
SOCIAL SUPPORT	
Families with limited English proficiency benefit from additional support from social work or peer-to-peer support programs as they prepare for discharge.	[12, 15]
PRIMARY CARE INVOLVEMENT	
A primary care provider should be chosen prior to discharge because culturally- and linguistically-appropriate options for a medical home may be more limited. If the family has not chosen a primary care provider, the NICU team can help them make this selection.	[1, 3, 6, 12]

PARENTAL MENTAL HEALTH	
Recognize that mental health issues can be more difficult to identify, monitor, and treat in families with cultural and linguistic differences. Any depression or mental health screening of parents must be translated and adapted so that it is culturally accurate and appropriate.	[15]

While in the NICU, families with limited English proficiency may experience more isolation. As much as possible, eliminate language as a barrier. Keep in mind that computer automated translation services often follow algorithms and lack the nuance and cultural context that may be provided by a certified medical interpreter.

These types of computer software and services are discouraged for any important medical discussion because they may not be linguistically or culturally accurate. The use of computer automated translation services should be limited to urgent situations when all other options have been exhausted or for very basic uncomplicated inconsequential information.

### Military families

This section focuses on discharge planning conducted with military families (see Table 15). The Department of Defense’s Exceptional Family Member Program (EFMP) is an invaluable resource for families who have children with special medical and developmental needs.

**Table 15:** Military families.

MILITARY FAMILIES	
RECOMMENDATIONS	SUPPORTING REFERENCES
FOLLOW UP CONSIDERATION	
In order to assess for appropriate community resources and follow-up care, ascertain where the family is currently living and where they are planning on living after discharge.	[32]
PRIMARY CARE CONTACT	
Plan for transition of care to a medical home team of providers, because military families may not have consistent providers for care.	[32]
DISCHARGE SUMMARY	
Provide family with a copy of the entire medical record to take to their next destination. If this is not possible, provide the family with copy of the discharge summary or information about how to obtain a copy of the discharge summary and/or medical record after discharge from the NICU.	[5, 32]
SUPPORT PROGRAMS	
If a family is military-connected, offer to use military resources available to family. To access military resources, contact Military OneSource at https://www.militaryonesource.mil or 800-342-9647.	[32]
Military chaplain care services are available via Military One Source’s extensive spiritual support networks.	[32]
The NICU discharge team should download and become familiar with the special needs toolkit, “Birth to 18.” It has valuable information for families navigating early intervention programs and special education services. It also has guidance on accessing TRICARE benefits, connecting with support services, and exploring opportunities for relocating to areas where appropriate services are available. https://download.militaryonesource.mil/12038/EFMP/PTK_SCORs/ParentToolkit_Apr2014.pdf.	[32]
The Exceptional Family Member Program (EFMP) is a program where civilian and military programs are coordinated to provide community support to families and help them meet their medical, educational, and housing needs. EFMP can help ensure that a family does not get moved to an area that doesn’t have the needed services.	[32]
The Educational and Developmental Intervention Services provides comprehensive developmental services, including early childhood special education, speech and language therapy, occupational therapy, physical therapy, social work support, and child psychology for families located overseas or at limited installations in the United States.	[32]
HOME VISITATIONS	
Remind active-duty military families that New Parent Support Program Home Visitation may be available for up to 36-48 months if the family resides near a military installation. (Families may obtain contact information through Military OneSource, 800-342-9647).	[32]
TRICARE INSURANCE	
• TRICARE Insurance is the healthcare insurance for active-duty and active-reserve military families: Information available at www.tricare.mil. • Defense Enrollment Eligibility Reporting System (DEERS) enrollment required within 30 days of the infant’s birth. • TRICARE will help a family find a provider who accepts their insurance. • TRICARE provides a nurse advice line. • Under TRICARE, many infants in active-duty families will be eligible for Extended Health Care Option for respite care, ABA clinical intervention, etc. Encourage family to discuss needs with a TRICARE representative. • TRICARE is not universally accepted. It can be a challenge to obtain services, especially mental health services. • If the infant of a medical family will require medical transport, coordinate with family’s medical insurance (TRICARE). Contact Relief Society for that service if transport causes financial distress for family. (The Relief Society for each branch of service may be obtained through Military OneSource, 800-342-9647).	[32]

Military families are not a homogenous group. For military families, there may be issues associated with frequent moves, including the following: change of medical providers/facilities, records of care kept in multiple locations, and access to services that varies by location. Some families are cared for in military hospitals. Others are cared for in civilian hospitals. Other families experience a hybrid of military and civilian hospital care. Military families in military facilities face similar issues as those in civilian facilities (e.g., deployment, frequent relocations, and heterogeneity of local resources).

Military medical facilities use the same electronic medical record. When a family is in military medical facilities, their records will be available because they follow them between military medical facilities. Military medical facilities have no access to medical records from civilian hospital unless the family brings/provides them. Therefore, when families are in civilian institutions, they must obtain and keep a copy of their records to provide to their military providers. Given this, there is potential for lack of transfer or loss of medical records when medical care is a hybrid between military and civilian facilities.

Some parents will be deployed and therefore not able to be physically present in the NICU. The following are helpful for deployed parents:

- During the NICU hospitalization, include the deployed parent as much as possible in the decision making.
- Use video conferencing - when possible - to allow a deployed parent to participate in discharge education.
- Define what conditions and circumstances would mandate bringing a deployed parent back from deployment.

Non-military individuals (e.g., extended family members, friends, clergy, etc.) may be allowed into military facilities to provide support to military families.

In life and death situations where a member of a military family is not present because they are deployed or stationed in another location, the Red Cross offers assistance. The Red Cross will confirm the dire prognosis directly with the medical team. Once the Red Cross has confirmation of the situation, they will reach out to the parents’ commanding officer to inquire about parental leave. The commanding officer will decide if they will grant the parental leave. If parental leave is granted, the Red Cross will help arrange and provide financial relief, if needed, for the transport.

### LGBTQIA+ headed families

LGBTQIA+ stands for lesbian, gay, bisexual, transgender, queer, intersex, and asexual. This section discusses important considerations for discharge planning with families that are LGBTQIA+ headed (see Table 16).

**Table 16:** LGBTQIA+ headed families.

LGBTQIA+ HEADED FAMILIES	
RECOMMENDATIONS	SUPPORTING REFERENCES
INCLUSIVE CULTURE	
Use gender-inclusive terms in all forms and teaching materials. For example, use terms like “caregiver” and "parent" instead of “mother” or “father.” Ask family members how they prefer to be addressed and referred to. Model respectful, inclusive, family-centered behavior.	
PARENTAL RIGHTS	
Make certain your NICU has clear policies and the appropriate forms to facilitate full legal access to infants, as requested by birth parents, non-gestational parents, adoptive parents, non-custodial parents, and legal guardians.	
Confirm all legal processes are in place to allow for Authorization to Consent as needed for alternate caregivers, including situations involving gestational carriers. Be familiar with your state laws for these situations and proactively have all legal processes in place.	
Involve all caregivers and, if applicable, legal guardians in discharge preparation and planning.	
Guarantee non-biological parent has authorization to consent through necessary legal forms and explain to non-biological primary caregivers the limitations of consent based on your state’s specific laws.	

### Parents with disabilities

Parents with disabilities may need accommodations to support and facilitate their full and active participation in discharge planning. This section discusses important considerations for collaborating with parents with disabilities during discharge planning (Table 17).

**Table 17:** Parents with disabilities.

PARENTS WITH DISABILITIES	
RECOMMENDATIONS	SUPPORTING REFERENCES
FAMILY LITERACY	
Ask parents with disabilities about their preferred method for communicating discharge information and honor those requests. For example, utilize multimedia and multimodal discharge teaching tools and instructions. Do not rely solely on written material to meet communication and health literacy needs. When possible, arrange for American Sign Language (ASL) or other interpreters in advance. Note that a parent in need of interpretation may wish to have a support person interpret instead of a staff interpreter.	[1, 5]
ACCESSIBILITY	
Parents with disabilities, such as those intellectual or developmental disabilities, may need extra time at discharge to have their questions answered.	
Parents with sensory sensitivities to noise or busy environments may wish to have the discharge discussions in a quieter place, such as a conference room.	
Ensure the facilities meet Americans with Disabilities Act (ADA) standards and that they can be modified to accommodate and support parents with disabilities. Make changes, with the parent’s input, as needed to support inclusion of all primary caregivers’ presence in both the patient’s room and any place where instruction is given or information is shared.	
Ask caregivers if they would like help from Adult Occupational and Physical Therapists in adapting caregiving tasks. This is also an appropriate time for OTs and PTs to evaluate any mobility aids that the parent may use and make changes to that equipment to make parenting tasks easier.	
HOME ENVIRONMENT	
Ask parents with disabilities if they need assistance with home modifications. Because states vary greatly in the home modification services they provide for people with disabilities, it is important to find out what home modification services are available where they live. Some people with disabilities are already living in appropriately accessible housing and may only need accessible baby equipment (e.g., wheelchair-accessible cribs or baby carriers). When the home environment needs to be modified to be accessible, inquire with your state’s Office of Disability Services (or other applicable agency) about funding options. For example, some states provide zero interest home modification loans for accessibility-related home modifications. Also consult with non-profit agencies and local advocacy groups (e.g., Parent-to-Parent, Easter Seals, United Way, etc.) to determine if funding is available to make needed changes within the home.	

CAREGIVER ABILITY	
Confirm that the caregiver is able to perform technical infant care skills on their own or with support. Ensure that there are sufficient supports in place for a safe discharge.	
Because the specialty care team providers can be a source of information about the parent’s abilities, consult with the specialty care team.	
Make sure lactation consultants and other support service providers have the needed skills, training, and expertise to work with parents with disabilities. Use ADA-certified specialists when clinically indicated.	[25]

Like any parents of preterm or medically-fragile babies, parents with disabilities may feel overwhelming emotions and need time to acclimate to the NICU environment. Additionally, they may be feeling extra pressure or stigmatization because of their disability status. It is important for providers to have awareness of their own biases about people with disabilities, and to not assume that deficits in caregiving ability are due to the parent’s disability status. Parents with disabilities will be most comfortable with providers whom they trust and who view them as competent and capable of taking care of their child (with or without supports). Talk to the parent about any barriers to infant care that you perceive and look for solutions together. A parent with disabilities’ ability to care for their infant should never be evaluated in an environment that is not fully accessible to them. This includes the presence of adaptive baby care equipment if needed. Ensure that parents with disabilities have sufficient space, time, and support to master technical infant care skills. Whenever possible, provide the most appropriate support. Ask parents with disabilities about their needs and preferences. Table 17.

Ask parents with disabilities about their accessibility needs. More often than not, people with disabilities are the experts about their own needs and conditions. Parents with disabilities often report that they are viewed as incompetent due to their disability status. It is imperative that we meet these parents where they are with their parenting skills and help them build self-efficacy, just as we would with parents without disabilities.

For some parents with disabilities, facilities that only meet minimal ADA standards may not adequately meet family’s needs. Whenever possible, work with the parents to find accessibility solutions that allow them to participate fully in their infant’s care and facilitate bonding, especially skin-to-skin care.

The National Research Center for Parents with Disabilities at Brandeis University https://heller.brandeis.edu/parents-with-disabilities) is an excellent resource for the current state of the science on disabled parenting for providers and resources for social work. The Center also produces numerous tip sheets, videos, and other informative resources for parents with disabilities.

No parent should fear seeking appropriate mental health care. But many do. Be aware that parents with visible disabilities may be reluctant to disclose “invisible disabilities” or mental health diagnoses (i.e., postpartum depression or anxiety) out of fear of further stigmatization. Mental health resources should be provided to these parents as well as assurances that they will be supported and affirmed when seeking appropriate care.

Like many parents, the parents with disabilities may have challenges with lactation, pumping, and breastfeeding. Furthermore, options for lactation support for parents with disabilities may be limited because not all lactation consultants have experience working with parents with disabilities. Breastfeeding, by design, is an inherently accessible way to feed an infant. Extra time may be needed to help the parent-infant dyad with positioning and latching, especially if the parent has fine motor disabilities. Explore alternate feeding positions and try as many positional aids (i.e., lactation pillows, slings) as possible and appropriate. The lactation consultant should take extra time to ensure that the family has pumping equipment that works for them. Pumping bras are excellent tools for accessible pumping.

### Families with distinct cultural and/or philosophical expectations

The standardized comprehensive discharge planning process must be individualized to the family. In order to meet the needs of the family, it is important to understand the cultural and philosophical expectations of the family. Never assume to know a family’s beliefs or values. Even within identified cultural groups there will be important, discernable differences. Therefore, it is imperative to have a culturally respectful approach that is individualized and informed by the family. If there are questions about what is and is not culturally-appropriate, ask the family. This section discusses a few items to take into account when doing discharge planning. Table 18.

**Table 18:** Families with distinct cultural and/or philosophical expectations.

FAMILIES WITH DISTINCT CULTURAL AND/OR PHILOSOPHICAL EXPECTATIONS	
RECOMMENDATIONS	SUPPORTING REFERENCES
FAMILY BELIEF SYSTEMS	
Belief systems (e.g., religion, faith, doctrine, philosophy, spirituality, etc.) shape a family’s medical experience. Ask how the NICU staff can be supportive and respectful of their belief systems.	[2, 8, 36]
Ask the family to share information about their belief systems including: • Specific cultural circumstances. • Religious and/or spiritual grounding. • Role of extended family. • Role of community, cultural, and spiritual leaders. • Any information the family feels is important to understand their unique needs. Document this information in the record.	[2, 6, 8, 15, 36]
Establish an environment that encourages questions and communication. Remember, every family’s NICU experience is unique, and each family may react to trauma differently.	
Designate a specific location in the medical record where relevant information the family shares can be found. This avoids the need for the family to repeatedly communicate information to multiple providers (excluding confidential information shared by a family member). Ask the NICU team members to review this section at the beginning of their shift and/or prior to interacting with the family.	
FAMILY SUPPORT PEOPLE	
Confirm with the family who they would like to have as their designated support network.	[2, 8]
Invite and encourage families to include a cultural or community leader to be present for teaching and at important meetings when it is the family’s preference. Do not wait for this request, ask if this is something the family would like to do upfront to support inclusiveness and enhance social support for the family.	[2, 15]
CULTURAL PRACTICE	
Work with the family to incorporate special cultural practices (e.g., a cornhusk bath), and adapt them if medically necessary. Utilize appropriate tools and technology to make these practices accessible and inclusive.	[2, 8, 36]
COMMUNITY RESOURCES	
When making a referral to community resources (e.g., early intervention, visiting nurse program, etc.) confirm with the family where they would like to receive their services. Services may be delivered in many settings, not just at home.	[3, 18]
Identify which community services and resources are available and appropriate for the family within the context of their cultural and philosophical belief systems.	[3, 8]
When indicated, discuss the need for visiting nurse or early intervention indicated services in advance and plan creative options when a family’s beliefs or circumstances do not allow outsiders in their home. Alternative meeting locations per the family’s request could include, for example, a local clinic, library, day care, mosque, church, temple, cultural center, etc.	

Ensure discharge education strategies are culturally-appropriate and tailored to meet the specific needs of the family. Supporting the family’s unique cultural philosophy and expectations is always important throughout the family’s stay in the NICU and when planning for discharge. Confirm that all written materials given to families is written in a manner that is simple, clear, concise, and devoid of medical jargon to aid understanding and decrease confusion. Be flexible with which caregivers are given the instruction (e.g., in some cultures it is the grandmother, not the mother, who primarily cares for the infant). Have the tenets of diversity inclusion and heath equity inform practice (e.g., https://diversityinformedtenets.org/about/the-tenets/).

Families should define their own support network. There can be cultural differences in parenting and caregiving roles. As part of the family assessment, it is important to ask about roles and expectations because otherwise this information may not be disclosed.

Some families will be reluctant to have an outsider enter their space. This should be taken into account when making arrangements for services such as visiting nurse or early intervention. Some services can be provided in a community setting (e.g., daycare, school, church, etc.) if the family prefers.

## Appendix

The NPA would like to thank the work group that convened to develop guidelines; the June 2019 and January 2021 national summit content experts who helped focus, revise, and review the guidelines; the five members of the work group affectionately referred to as the “small group” who verified the references; and specifically, Patti Bridges, Brigitte C. Desport, and Julia Yeary for their careful review and thoughtful editorial support.

**Organizer**

National Perinatal Association

**Chairperson**

Vincent C. Smith, MD, MPH

**Steering Committee**

Erin Armknecht, BA

Patti Bridges, MSW, LCSW

Joy Browne, PhD, PCNS, IMH-E (IV)

Jenene Craig PhD, MBA, OTR/L

Brigitte C. Desport, DPS, OTR/L, BCP, ATP

Erika Goyer, BA

Cristal Grogan

Andrea Werner Insoft, LICSW, ACSW

Kristin Love

Cheryl Milford, EdS

Steve Richardson

Tiffany Willis, PsyD

Julia Yeary, LCSW, IMH-E®

**Interdisciplinary Guidelines and Recommendations Development Workgroup**

Erin Armknecht, BA

Patti Bridges, MSW, LCSW

Joy Browne, PhD, PCNS, IMH-E (IV)

Jenene Craig PhD, MBA, OTR/L

Brigitte C. Desport, DPS, OTR/L, BCP, ATP

Heidi Gates, RN

Erika Goyer, BA

Cristal Grogan

Andrea Werner Insoft, LICSW, ACSW

Carol Jaeger, DNP, RN

Judi Kleekamp, PT

Kristin Love

Cheryl Milford, EdS

Trudi N. Murch, PhD, CCC-SLP

Heather Cohen Padratzik, MHA, JD

Steve Richardson

Cuyler Romeo, MOT, OTR, SCFES, CLC

Betty Vohr, MD

Tiffany Willis, PsyD

Julia Yeary, LCSW, IMH-E®

**Additional Content Expert Contributors**

Michael T. Hynan, PhD

Carole Kenner, PhD, RN, FAAN

Jonathan S. Litt, MD, MPH, ScD

Nicole Lomerson, MPH

Molly Fraust Wylie

**NPA Staff**

Erika Goyer, BA

Kristin Love
